# The Pedicled Flap of Adductor Longus, a New Technique for Inguinal Reconstruction

**DOI:** 10.3389/fsurg.2021.639893

**Published:** 2021-12-14

**Authors:** Hong Zhang, Zhenfeng Li, Jianmin Li, Lei Zhu, Yakubu Ibrahim

**Affiliations:** ^1^Department of Orthopedics, Qilu Hospital of Shandong University, Jinan, China; ^2^Cheeloo College of Medicine, Shandong University, Jinan, China

**Keywords:** adductor longus, inguinal region, reconstruction, radical local resection, inguinal lymph node dissection

## Abstract

**Introduction:** Reconstruction surgeries of the inguinal area pose a challenge for oncological and orthopedic surgeons, especially after radical local resection (RLR), radical inguinal lymph node dissection (RILND), or both. Although numerous surgical procedures have been reported, there is no report about a pedicle adductor longus flap method. The aim of this work is to show our experience about inguinal reconstruction with pedicled adductor longus flap and associated outcomes.

**Patients and Methods:** A retrospective study of 16 patients with localized inguinal region interventions and reconstructed by adductor longus flap from March 2016 to July 2020. Patients' average age was 60.0 years (range = 38–79 years) and had postoperative follow-up of 10 months (ranging 2–19 months). All patients had unilateral inguinal region involvement—seven cases on the left and nine cases on the right. The patients' clinical course, operative course, and postoperative follow-up data were evaluated.

**Results:** All 16 patients recovered well post-operatively and did not require any re-intervention. Four patients experienced negligible discomfort around the groin area. Five patients experienced a minor strength deficit in thigh adduction compared with that of preoperative strength in the same or contralateral leg. The aforementioned complications resolved during the postoperative course and had no functional impact on their activity of daily living. All adductor longus flaps survived, completely filled the inguinal dead space, and wounds healed uneventfully within 3 weeks except for three patients who suffered delayed wound healing for more than 4 weeks. Other common complications such as infection, seroma, or wound dehiscence were not encountered in this series.

**Conclusion:** The adductor longus flap is a reliable alternative method for inguinal region reconstruction following radical local resection (RLR), radical inguinal lymph node dissection (RILND), or both.

## Introduction

The inguinal region is a crucial intersection of fundamental anatomical structures, such as the femoral artery, vein, nerve, the inguinal node stations, and the inguinal canal. This makes the inguinal region a common surgical site for many different interventions of different departments, in which the surgical lymphadenectomy and diverse oncological resections are the most common. These may result in soft tissue defects and exposure of vital anatomic structures in this region. Direct closure without any reconstructions for this region is more likely to produce a primary soft tissue defect or dead space, leading to wound dehiscence, delayed healing, and postoperative abscess formation. The anatomical features of inguinal defects between the abdomen and the thigh make the reconstruction of the inguinal region challenging for surgeons. The poor wound healing in the inguinal region has been attributed to wide defects with bacterial contamination, non-collapsible dead spaces, lymphatic leaks, and the healing difficulties related to a low vascularized or eventually irradiated field, depending on the primary pathology (1, 2). The postoperative morbidity related to inguinal surgeries documented in the literature indicate a high incidence rate of complications of 40% (3).

Various reconstruction techniques have been reported for the inguinal region. Besides myocutaneous flaps and fasciocutaneous flaps, the most common used muscle flaps are sartorius flap(S-M), rectus abdominis flap (RA-M), tensor fascia lata flap (TFL-M), gracilis flap(G-M), and rectus femoris flap (RF-M) (2, 4–10). However, there is no report on pedicled adductor longus flap for the reconstruction.

This study aims to share our experience of using adductor longus flap as a muscle-only pedicled flap for reconstruction of the inguinal region after diverse oncological resections, lymphadenectomy, or both simultaneously, and presents pertinent real-world cases of its applications as a tool for orthoplastic reconstruction.

## Patients and Methods

### Data Collection

From March 2016 to July 2020, 16 patients (5 males and 11 females) treated with pedicled adductor longus flaps for the defects of inguinal region following radical local resection (RLR), radical inguinal lymph node dissection (RILND), or both were retrospectively selected for the study. Patients' average age was 60.0 years (range = 38–79 years) and had postoperative follow-up of 10 months (ranging 2–19 months). All patients had unilateral inguinal region involvement—seven cases on the left and nine cases on the right. The patients' clinical course, operative course, and postoperative follow-up data were evaluated. The patients in this series had on average 0.5 significant comorbidities (range, 0–2), namely, hypertension (*n* = 5 patients), diabetes (*n* = 2), coronary artery disease (*n* = 1). The demographic and clinical details of the patients are shown in Table 1. This study was approved by Medical Ethics Committee of Qilu Hospital of Shandong University. However, due to the retrospective nature of the study, a formal patient's consent was not required.

**Table 1:** Clinical data.

**Patient No**.	**Age(y)/Sex**	**Diagnosis**	**Comorbidities**	**Side L(eft)/R(ight)**	**Operation**	**Follow-up(month)**	**Complication**
1	65/F	FS	HP	L	RLR, RILND	6	Healed, None
2	68/F	Lymphadenectasis	None	L	RILND	7	Healed, None
3	49/M	Lymphoma	None	L	RILND	11	Healed, None, discomfort around the groin, strength deficit of thigh adduction
4	58/M	Lymphoma	None	L	RILND	10	Healed, None
5	39/F	Cyst	None	R	RLR	14	Healed, None, discomfort around the groin, strength deficit of thigh adduction
6	75/F	SS	HP	R	RLR, RILND	6	Delayed Healed, None
7	60/F	MM	HP, DM,	L	RILND	6	Healed, None
8	70/F	MM	None	L	RILND	6	Healed, None
9	79/M	FS	DM	R	RLR, RILND	12	Delayed Healed, None
10	73/M	MM	HP	R	RILND	6	Delayed Healed, None
11	59/F	LGFMS	HP, CHD	R	RLR, RILND	15	Healed, None
12	38/F	DTSGC	None	L	RLR	19	Healed, None, discomfort around the groin, strength deficit of thigh adduction
13	58/F	MM	None	R	RILND	10	Healed, None
14	74/M	Cyst	None	L	RLR	16	Healed, None
15	47/F	LGFMS	None	L	RLR, RILND	15	Healed, None, strength deficit of thigh adduction
16	49/F	Liomyoma	None	R	RLR, RILND	2	Healed, None, discomfort around the groin, strength deficit of thigh adduction

*(FS, Fibrosarcoma; SS, synovial sarcoma; MM, Melanoma; LGFMS, low-grade fibromyxoid sarcoma; DTSGCT < diffuse tenosynovial giant cell tumor; HP, hypertension; DM, diabetes mellitus; CHD, coronary heart disease; RLR, radical local resection; RILND, radical inguinal lymph nodes dissection)*.

### Surgery Procedures

**Figure 1:**
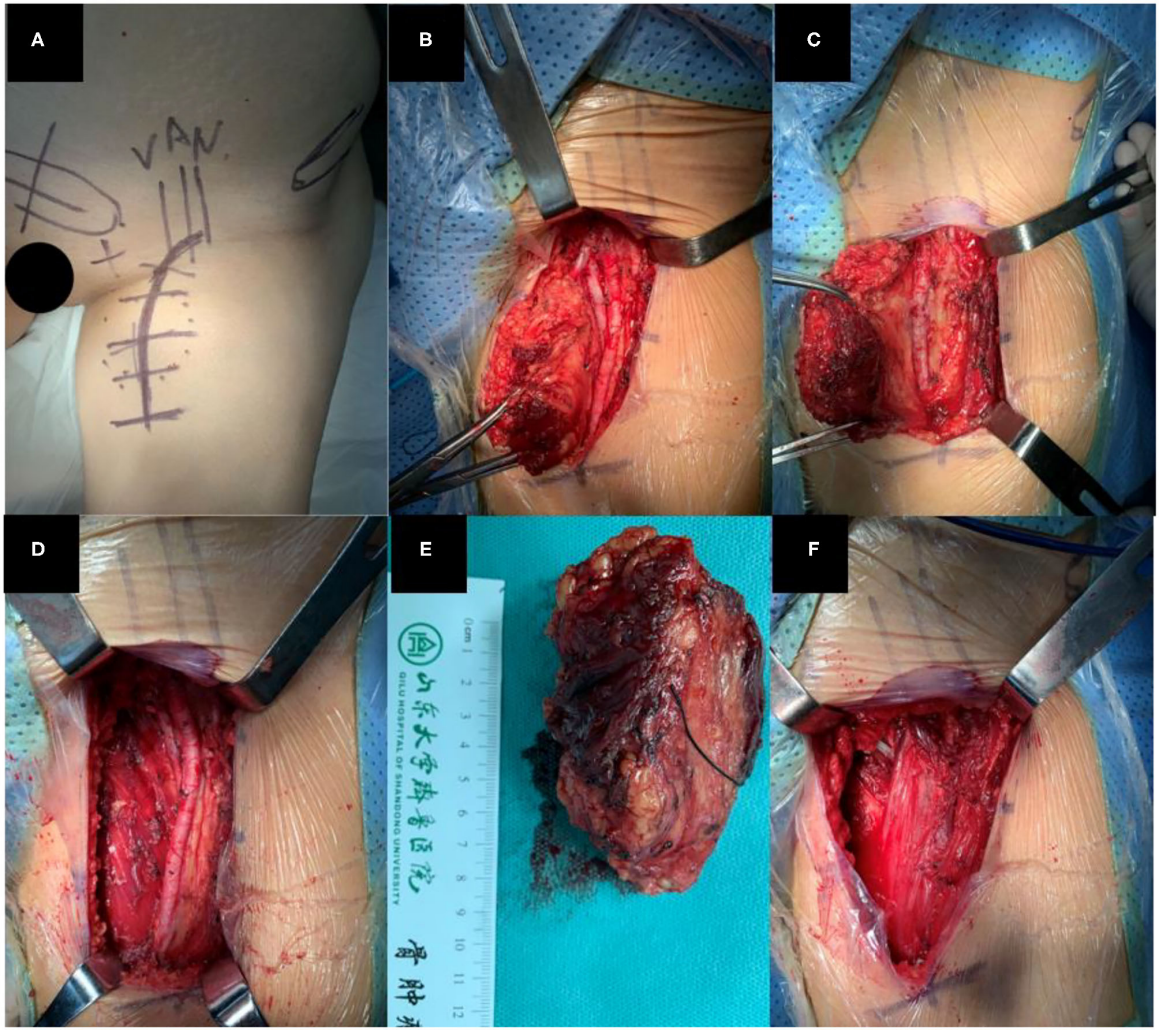
**(A)** Incision design before surgery, **(B,C)** Resection of tumor, **(D)** After local wide resection **(E)** Resected tumor, **(F)** After adductor longus reconstruction.

**Figure 2:**
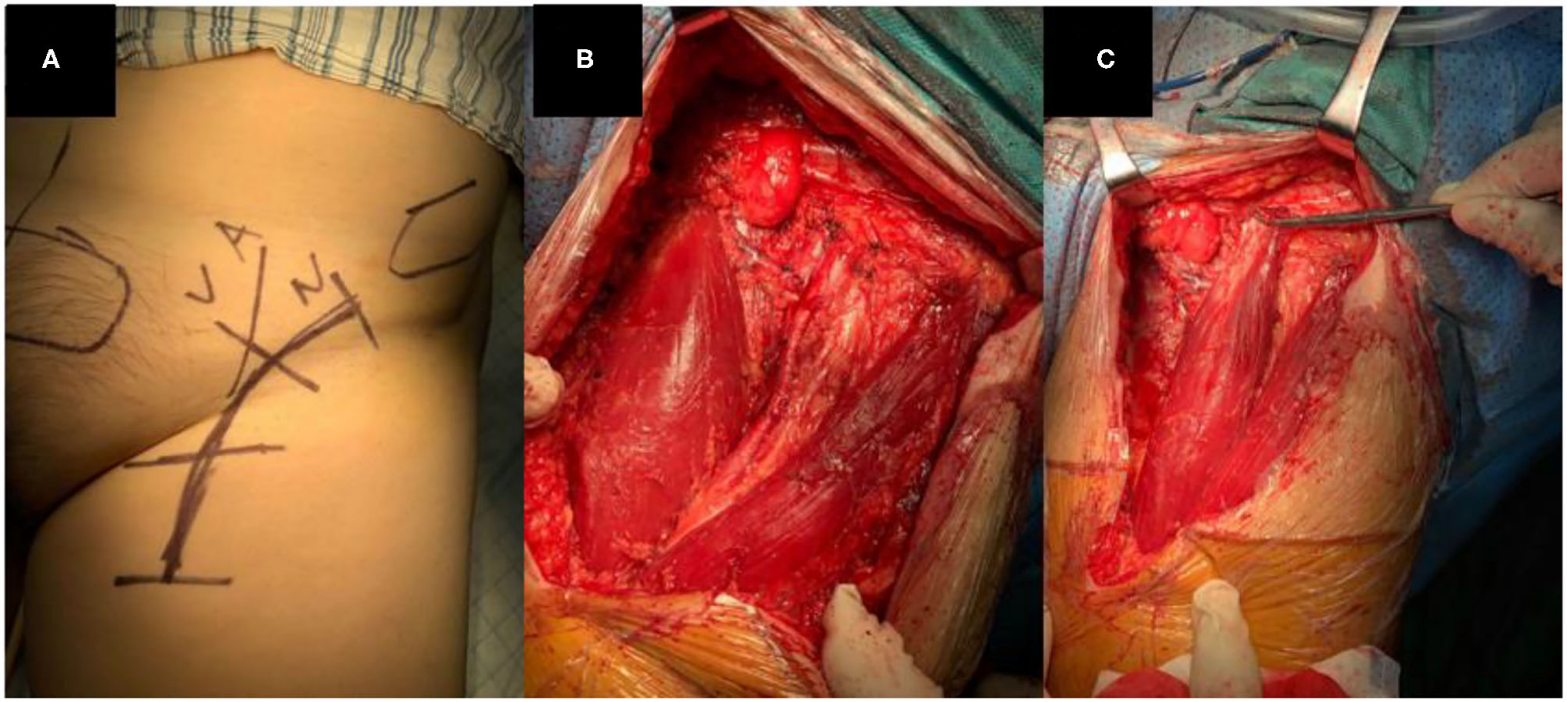
**(A)** Incision design before surgery, **(B)** After lymphadenectomy and adductor longus in the medial side of incision, **(C)** After adductor longus reconstruction, the muscle with the clamp is the adductor longus flap.

The patient was in supine position with the leg abducted after general anesthesia. An arc-shaped incision which started from nearly 2–3 cm below the anterior-superior spine to the inner thigh was made (Figures 1, 2). Once the femoral triangle was exposed, the saphenous vein was ligated proximally at the saphenofemoral junction, and distally at the apex of femoral triangle. The femoral canal contents were identified and preserved. Then, we carried out radical local resection (RLR), radical inguinal lymph node dissection (RILND), or both of them around the ilioinguinal region. The proximal insertion of the adductor longus was exposed and dissected. The severed adductor longus muscle flap was carefully peeled to the distal angle of the femoral triangle. The peeling of the flap was performed with caution to preserve the major muscular perforators and branches. The flap was then transposed to cover the femoral vessels and the detached origin was anchored to the inguinal ligament above the femoral neurovascular bundle with 4–5 interrupted stitches, using the size 0 absorbable suture (Figures 1, 2).

**Figure 3:**
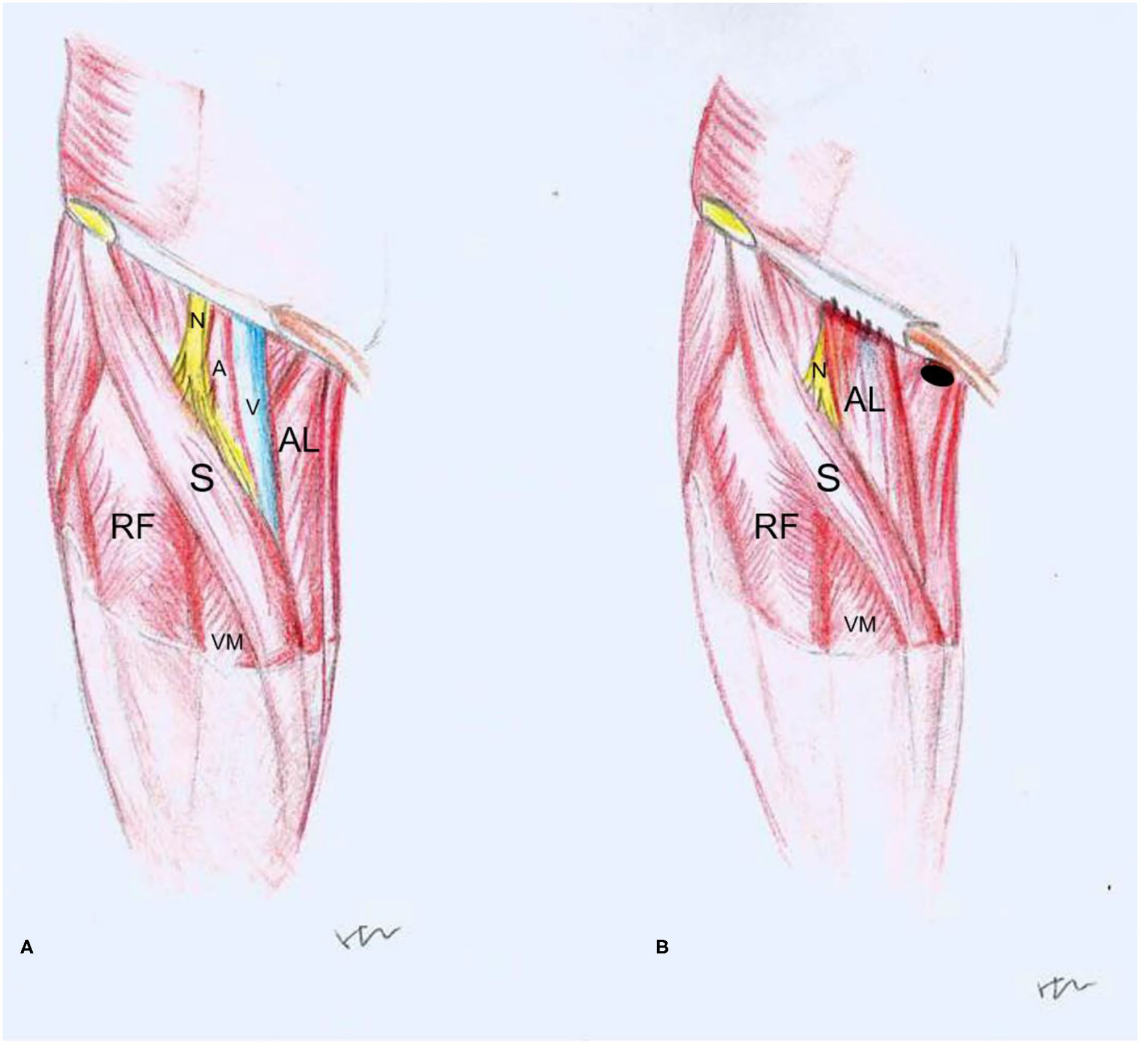
Drawings before **(A)** and after **(B)** reconstruction with adductor longus flap (RF, rectus femoris; AL, adductor longus; S, sartorius; VM, vastus medialis; N, femoral nerve; A, femoral artery; V, femoral vein, •: adductor longus attachment of pubis).

The flap creation before and after harvesting is shown in Figure 3. The defect of the inguinal area ranged from 7 × 4 to 9 × 6 cm and was filled with this muscle flap. A suction drain was placed on the wound and was kept in place until the volume drained was below 15 ml per day. The incision was closed in layers.

### Postoperative Course

The patients underwent stitch removal at 3 weeks after surgery and initiated mild to moderate physical activities without weightbearing on the operated limb for 4 weeks. Between 4 and 12 weeks postoperatively, a guided physical activity was encouraged which focused on return of full motion, strengthening, and return to a normal activity of daily life.

## Results

The mean hospital stay was 10 days postoperatively. The employed adductor longus flap transposition method successfully covered the femoral vessels and obliterated the inguinal dead space.

All patients had a satisfactory postoperative recovery without any major complications. However, four patients (three females and one male) complained of a negligible non-specific discomfort around the groin area. A mild adduction strength deficit in the operated thigh was noticed postoperatively in five patients (four females and one male). On examination, the preoperative and postoperative adduction muscle strength of the four female patients decreased from IV+ to IV–, respectively, and the male patient's strength was reduced fromV to IV–. The strength deficit, which resolved during the postoperative course, could be attributed to the adductor longus muscle resection and its transposition. All wounds healed uneventfully in all patients within 3 weeks, except for three patients who had delayed would healing (more than 4 weeks, postoperatively). None of the patients required re-intervention and there were no signs of infections, seroma, wound dehiscence, flap loss, partial flap necrosis, hematoma formation, or venous congestion. Other potential complications indirectly involving the flap, such as abdominal wall hernia and lymphedema of the lower limbs, were not observed.

## Discussion

Reconstruction surgeries involving the groin can be challenging for both oncological and orthoplastic surgeons. The incidence of wound complications after radical inguinal lymph node dissection (RILND) has been reported to be ranging from 14 to 77% (11–16).

Many reconstruction procedures, including myocutaneous flaps, fasciocutaneous flaps, and muscle flaps have been reported during the last two decades (4). Pedicled muscle flaps reported in literatures are sartorius flap(S-M), rectus abdominis flap (RA-M), tensor fascia lata flap (TFL-M), gracilis flap(G-M), and rectus femoris flap (RF-M) (2, 4–10). Because the S-M is a type IV muscle, which has a series of segmental pedicles for blood supply (17), some surgeons are warry about the flap loss after transportation. The RA-M may weaken the abdominal wall resulted in abdominal hernia (18–20). G-M is a trap muscle which has a lower amount of muscle volume, comparatively. On the other hand, Naveet Kour et al. concluded in their cadaveric studies that the adductor longus is a good candidate for functioning free muscle transplantation based on its single vascular anastomosis (21). However, a report about adductor longus for real-world reconstruction is not yet published.

Adductor longus is one of the medial group muscles of thigh. It is a long, slender, triangular muscle, which originates from the pubic body and inserts onto the middle of the linea aspera of femur with good excursion (17). It contributes to adduction, flexion, and lateral rotation of the thigh, together with the other four muscles of medial group. Compared to the sartorius, the gracilis, and rectus abdominis, it has a better muscle volume.

In the current study, four patients complained of non-specific discomfort around the groin area and five patients experienced a mild thigh adduction strength deficit postoperatively. These patients belonged to the younger group in the study. For younger patients who have a higher demand for athletic ability, this muscle flap may result in some discomfort and a minor strength deficit in thigh adduction. Furthermore, Linnea Welton et al. reported case series of 19 National Football League players who suffered adductor longus rupture (21). On examination, patients had painless range of motion at the hip and knee. However, pain was reproduced with resisted thigh adduction (22–28). This suggests that a strength deficit may be encountered in patients with adductor longus muscle injury (29–32). Similar studies showed that the primary function of the adductor longus is as a stabilizer and not for producing power during cutting maneuvers (32). It is minimally active during in-line jogging and sprinting (33). This explains why the adductor longus flap creation may lower the strength of thigh adduction with discomfort that is exacerbated with demanding physical activities. This is consistent with what was observed in the current study, suggesting that reconstruction with adductor longus flap is maybe a perfect alternative in elderly patients with malignant tumors in this region. The patients who experienced strength deficit in this series were relatively younger, with an average age of 44.4 (from 38 to 49), than those who did not have such deficit. The caveat to this approach, as observed, was that it weakens the medial edge support function of the femoral triangle which may lead to muscle strength deficit, especially in active younger patients. Therefore, expertise, firm repair, and anchoring of the adductor longus are essential when applying the method described in this study for an optimal clinical outcome.

Wang J et al. revealed that the adductor longus muscle was innervated only by the obturator nerve in a cadaver study. Also, the adductor longus muscle nerve branch entered at the superolateral deep surfaces of the muscle and divided into two primary nerve branches that extended from the superolateral to the inferomedial side. The nerve distribution of the inferolateral and superomedial part of the muscle is sparse. (34) The likelihood of the nerve damage in a case of slight displacement during adductor longus tagging is minimal. According to Wang ZT et al., the intramuscular branches of the muscle comes from the femoral artery (35), and is far enough from the femoral triangle to prevent its involvement in the primary lesion. The adductor longus flaps reconstruction method employed in this study has lesser injury risk to the perforator and the adductor longus muscle innervation structures.

Limitations of the current study include its small sample size, retrospective nature, lack of control group, and that it is not randomized. However, the feasibility and the overall outcome of this method is reliable. The adductor longus method applied here is being reported, according to our knowledge, for the first time and it demonstrated that the adductor longus flaps have a smaller displacement and provide a better fill effect than either the sartorius or the gracilis. A rigorous dissection is not necessary for harvesting and transposition of adductor longus and hence, lowering the risk of disrupting the blood supply to the skin of the inguinal region. The smaller displacement of muscle, the less functional impact. The adductor longus flap creation moves the medial group muscle to the femoral triangle, balancing the soft tissue volume, and making it impossible to form a dead space, postoperatively. The pedicled adductor longus flap is an available technique in inguinal region reconstruction, especially when sartorius not available, with greater volume to replace and after resections of malignant tumors or inguinal lymph node dissections.

## Conclusion

The pedicled adductor longus flap is a promising additional flap choice for inguinal region reconstruction, especially for patients suffering malignant tumor around this area. It has low donor-site morbidity, little functional impact, is easy to harvest, and provides more muscle volume to fill up the potential dead space. We will further explore the vascular anatomy of the adductor longus flap and the prospective comparative study with other flap techniques in our future work.

## Data Availability Statement

The original contributions presented in the study are included in the article/supplementary material, further inquiries can be directed to the corresponding author/s.

## Ethics Statement

The studies involving human participants were reviewed and approved by Medical Ethics Committee of Qilu Hospital of Shandong University. Written informed consent for participation was not required for this study in accordance with the national legislation and the institutional requirements.

## Author Contributions

JL, ZL, and LZ made substantial contributions to the study conception and design. ZL, HZ, and YI made primary contributions to acquisition of data, analysis, and interpretation. HZ did the hand drawings of the Figure 3. ZL, HZ, and YI drafted the manuscript. All authors contributed to the revision of the manuscript.

## Conflict of Interest

The authors declare that the research was conducted in the absence of any commercial or financial relationships that could be construed as a potential conflict of interest.

## Publisher's Note

All claims expressed in this article are solely those of the authors and do not necessarily represent those of their affiliated organizations, or those of the publisher, the editors and the reviewers. Any product that may be evaluated in this article, or claim that may be made by its manufacturer, is not guaranteed or endorsed by the publisher.
